# Clinically sufficient classification accuracy and key predictors of treatment failure in a randomized controlled trial of Internet-delivered Cognitive Behavior Therapy for Insomnia

**DOI:** 10.1016/j.invent.2022.100554

**Published:** 2022-06-25

**Authors:** Erik Forsell, Susanna Jernelöv, Kerstin Blom, Viktor Kaldo

**Affiliations:** a Centre for Psychiatry Research, Department of Clinical Neuroscience, Karolinska Institutet, Stockholm Health Care Services, Stockholm County Council, Sweden; b Division of Psychology, Department of Clinical Neuroscience, Karolinska Institutet, Stockholm, Sweden; c Department of Psychology, Faculty of Health and Life Sciences, Linnaeus University, Växjö, Sweden

**Keywords:** Insomnia, Personalized medicine, Adaptive treatment strategy, Prediction, Internet-delivered Cognitive Behavior Therapy

## Abstract

**Background:**

In Adaptive Treatment Strategies, each patient's outcome is predicted early in treatment, and treatment is adapted for those at risk of failure. It is unclear what minimum accuracy is needed for a classifier to be clinically useful. This study aimed to establish a empirically supported benchmark accuracy for an Adaptive Treatment Strategy and explore the relative value of input predictors.

**Method:**

Predictions from 200 patients receiving Internet-delivered cognitive-behavioral therapy in an RCT was analyzed. Correlation and logistic regression was used to explore all included predictors and the predictive capacity of different models.

**Results:**

The classifier had a Balanced accuracy of 67 %. Eleven out of the 21 predictors correlated significantly with Failure. A model using all predictors explained 56 % of the outcome variance, and simpler models between 16 and 47 %. Important predictors were patient rated stress, treatment credibility, depression change, and insomnia symptoms at week 3 as well as clinician rated attitudes towards homework and sleep medication.

**Conclusions:**

The accuracy (67 %) found in this study sets a minimum benchmark for when prediction accuracy could be clinically useful. Key predictive factors were mainly related to insomnia, depression or treatment involvement. Simpler predictive models showed some promise and should be developed further, possibly using machine learning methods.

## Highlights

• Individual predictions enable use of adaptive treatment strategies for patients who risk not benefitting from treatment.
• We suggest a benchmark of a balanced accuracy of 67 % for predictions to be clinically useful to guide treatment adaptation.
• We examine 21 expert selected predictors and finds that only haft of them have a meaningful association with outcome.
• Severity on one hand and adherence and compliance on the other hand were the most important predictors of treatment failure.

## 1 Introduction^1^

Cognitive Behavior Therapy (CBT) is efficacious for a wide arrange of disorders and can be successfully delivered face-to-face, or via the internet (Carlbring et al., 2018; Cuijpers et al., 2019; Seyffert et al., 2016; van Straten et al., 2018). Yet, 25–65 % of patients do not achieve a satisfactory treatment outcome regardless of delivery format and type of disorders (G. Andersson et al., 2019; Lambert, 2015; Rozental et al., 2019; Slade et al., 2008), and this is also true for CBT for insomnia (Forsell et al., 2019b; Jernelov et al., 2012; Charles M. Morin et al., 2009; Ritterband et al., 2017).

Stepped Care, where patients start with low intensity/high availability treatment and moves up sequentially to more intensive treatments if needed, could reduce waiting times, costs, and the demand for qualified therapists (Bower and Gilbody, 2005) and provide a clear course of action for patients with an initial unsatisfactory treatment response. There is evidence suggesting that treatment effects at the end of a stepped care service can be non-inferior compared to starting with the more intense treatment right away (Mohr et al., 2019). This implies that despite many patients in stepped care receive less intensive treatment (i.e. stopping at a lower step) the overall effects are similar to what would happen if everyone would receive the most intensive treatment.

A drawback of stepped care could be that those who need treatment that is more intensive must go through, and fail, one or several treatments before they reach a treatment of sufficient intensity, which prolongs their suffering. For example, Mohr et al. (2019) found that patients in stepped care were less satisfied with their care than those who received more intensive treatment right away. An ideal way to avoid this drawback is through precision care, where patients are matched to the optimal intervention right away based on pre-treatment predictors (Goldberger and Buxton, 2013; Hallgren et al., 2017). However, within CBT and ICBT (internet-delivered CBT), predictors with sufficient strength to directly inform clinical decisions for an individual patient have yet to be identified (Gerhard Andersson, 2018).

Meanwhile, there is ample evidence that monitoring of symptoms and other factors early on in treatment improves our ability to predict how a patient will fare by the end of treatment (Forsell et al., 2019, Forsell et al., 2019; Hannan et al., 2005; Lambert, 2015; Lutz et al., 2017; Schibbye et al., 2014; Schueller et al., 2015). Therefore, an alternative or complement to stepped care might be early identification of patients who will not benefit from their ongoing treatment, and immediately intensify or adjust their treatment, instead of abandoning it for another treatment. This concept is referred to as an ‘Adaptive Treatment Strategy’ (Forsell et al., 2019b).

A key question when using an adaptive treatment strategy is how accurate the prediction needs to be before one can act upon it. So far, in relation to medical care, Eisenberg and Hershey (1983) found that clinicians were willing to act on predictions once they became about 65 % likely to be correct. However, this is a very preliminary and quite dated finding and is based on clinicians deciding on treatment, further testing or waiting in a wide range of situations quite dissimilar to psychotherapy or psychiatry (for instance cancer treatment, taking biopsies etc.). As such, there is an important knowledge gap where we have no clear benchmark for how accurate a classifier needs to be in order to be clinically useful in our field.

In a previous study, a proof-of-concept for an Adaptive Treatment Strategy in therapist-guided Internet-delivered Cognitive Behavior Therapy for Insomnia (ICBT-i) was presented (Forsell et al., 2019b). Patients were classified as at-risk of treatment failure or not, during the fourth week of treatment. After the classification, at-risk patients were randomized to either adapted treatment or to continue treatment as before. Results showed that treatment failure could be predicted and, if therapists intervened with a structured adaptation plan, a meaningful proportion of predicted failures could be avoided. The Odds ratio for failure for not-at-risk patients compared to at-risk patients who did not get adapted treatment was 0.17 (*p* < .001, whereas the Odds ratio for failure between not-at-risk patients and those at-risk patients who did get adapted treatment was 0.51 (ns). It can be argued that the classification algorithm in the proof-of-concept trial proved to be clinically useful and therefore accurate enough for this application, since the results of the trial was positive (Forsell et al., 2019b). However, a test of the classification accuracy and an in-depth analysis of the classification algorithm and its constituent predictors has not yet been performed. Doing this could establish an empirically supported minimal level of accuracy (good enough, benchmark) to use in an Adaptive Treatment Strategy. Examination of each predictor used by the classifier separately, could help identify potential improvements or simplifications for future research. Examining the individual predictors could also more specifically inform our understanding of the treatment process in CBT for insomnia.

## 2 Aims

In this study, we want to suggest a minimal empirically supported level of accuracy for a classifier to have to be clinically useful in an adaptive treatment strategy. Also, we want to explore and compare prediction models of different complexity and implementation potential. To achieve this, we have three specific aims:

1) to establish the accuracy of the RCT-classifier used to predict treatment failure in the previous proof-of-concept study (Forsell et al., 2019b), thereby creating a benchmark for empirically supported minimal accuracy in future developments of Adaptive Treatment Strategies
2) to examine the relative value of each of the predictors for the classifier, and
3) to examine the additive value of different logical sets of predictors.

## 3 Method

Data for the present study comes from a published randomized controlled trial (Forsell et al., 2019b), where patients undergoing ICBT-i were classified as Red (risk of failed treatment) or Green (not at risk) during treatment week 4 out of 9. For the sake of brevity and clarity, we will use the terms Green for patients who were predicted to have a good outcome (treatment Success) by the algorithm and Red for patients who were predicted to have a bad outcome (treatment Failure) by the algorithm. Please see Section 3.3 for definitions of Success and Failure.

The reason for making the classification at week 4 was a combination of two factors. 1) This is the point where patients working at the prescribed pace will have gone through all the psychoeducation and rationale and initiated sleep restriction (i.e. here we can see clearly who is falling behind, who is rejecting the rationale and who is having problems calculating sleep windows etc.) and 2) We still wanted as much time left in treatment as possible to adapt treatment and help those who we believed would not benefit enough with their current trajectory.

Half of the Red patients were randomly assigned to receive an adapted treatment. In the current study, we only examine data from all of the Green patients (*n* = 149) and those Red patients who did *not* receive adapted treatment (*n* = 51), in order to assess the accuracy of the classifier without the treatment adaption acting as a confounder. The 200 patients included in this study (149 Green +51 Red) have received the same level of care in the same treatment. The study was approved by the Regional Ethics Board in Stockholm, Sweden and was pre-registered at clinicaltrials.gov (NCT01663844.).

### 3.1 Participants and treatment procedure for the RCT

A detailed description of the intake, assessments, treatment and study procedures has already been published (Forsell et al., 2019b), but the trial methods are briefly described below.

The study was set at the Internet Psychiatry Clinic, a psychiatric specialist care clinic within public health care in Sweden. Patients self-referred to the study by filling out screening questionnaires on the official website of the Internet Psychiatry Clinic. A face-to-face psychiatric assessment including the MINI psychiatric interview (Sheehan et al., 1998) was conducted before inclusion. The inclusion criteria were: 18 years old or above, Insomnia diagnosis according to DSM-5 (American Psychiatric Association, 2013) and >10 points on the Insomnia Severity Index (ISI)(C. M. Morin et al., 2011), no changes in antidepressant use/no use during last 2 months, no night shift work, proficient in Swedish, no depression (patients with comorbid depression were included in another parallell trial), no diseases, disorders, or substance abuse that required other, immediate attention (e.g. suicidality) or that was considered a contraindication for the treatment in the present trial (e.g., sleep apnea). The treatment was a 9-week ICBT-i treatment focusing mainly on sleep restriction and stimulus control but also including other standard CBT-i interventions, such as cognitive reappraisal and relaxation exercises. At the time of classification, i.e. during treatment week 4, patients adhering to the treatment plan should have initiated sleep restriction. An overview of the treatment is presented in the Appendix.

### 3.2 The classification algorithm

#### 3.2.1 The creation of the classification algorithm

The classification algorithm was built prior to initiating the RCT by authors VK, KB and SJ. All input predictors and their weights are visible in a published Excel-spreadsheet (supplemental materials to Forsell et al., 2019b). The patient-rated predictors for the algorithm were chosen based which items from previous trials had the strongest association with outcome (Success/Failure) in regression and ROC-curve analyses using data from three older published trials (Blom et al., 2015; Jernelov et al., 2012; Kaldo et al., 2015a), as well as theoretical and clinical assumptions where no empirical cutoffs were available or could be calculated (for example that it should at least theoretically be a bad sign if the patient reports not having learned anything new about their condition during the initial treatment phase, even if no study to date has examined that). Self-rated predictors were kept to a minimum for the sake of the patients. For the clinician-rated part, predictors were selected based on the authors' experience and an overview of the field of CBT-i to inform each factor's relevance for outcome (e.g. initiating Sleep Restriction Therapy early and being active in treatment are likely to be important). The patient-rated measures that were included in the algorithm, as well as their measurement time points and references for the scales can be found in Table 1.

**Table 1:** Patient rated measures included in the RCT-classification algorithm.

Measure (abv.)	Measurement time points	Classification step	Reference
Insomnia Severity Index (ISI) sum week 3	Baseline + Weekly	2	Bastien et al. (2001)
Montgomery-Åsberg Depression Rating Scale-Self-report (MADRS-S) mean of Baseline through Week 3 & change Baseline to Week 3	Baseline + Weekly	2	Fantino and Moore (2009), Montgomery and Asberg (1979)
Clinical Outcomes in Routine Evaluation system-10 minus suicidality-item (CORE-9)	Baseline	2	Barkham et al. (2013)
Dysfunctional Beliefs About Sleep (DBAS) items 4,11,17,20,24,25,28, 29	Baseline	2	Espie et al. (2000)
“To what extent have the things you've read about and have done in treatment so far affected your knowledge about, and the way you think about, sleep and insomnia?”(5-point likert scale from Not at all to Very much) mean of three ratings	Weekly	2	
General Self Efficacy scale (GSE)	Baseline	3	Schwarzer and Jerusalem (1995)
Sleep Related Behaviors Questionnaire (SRBQ) items 7,9,25, 28	Baseline	3	Ree and Harvey (2012)
Sleep Problems Acceptance Questionnaire (SPAQ) items 1,2,3,4 and the additional item”There are many activities that I do even when I have slept poorly”	Baseline	3	Bothelius et al. (2015)
Perceived Stress Scale 4 items (PSS-4)	Baseline	3	Herrero and Meneses (2006), Warttig et al. (2013)
Working Alliance Inventory (short form, WAI) mean	Week 3	3	Falkenström et al. (2015)
Treatment Credibility Scale (TCS)	Week 3	3	Devilly and Borkovec (2000)

#### 3.2.2 The classification algorithm and procedure

The classification algorithm itself was contained in an Excel spreadsheet and had four steps that the clinician went through, stopping whenever a final decision could be made.

Step 1 was to sort out treatment dropouts and handle discrepant or obvious cases of classification. Patients who had ended treatment or wanted to leave the study before week four were not classified, but instead excluded. In some cases, the clinician could indicate that the available data was broken or untrustworthy (based on communication with the patient) and therefore deem using the algorithm invalid. The clinician and supervisor would then give an immediate classification of Red or Green without going further with the algorithm. This was rare (*n* = 5) and very much a last resort, but still counts as those patients' de facto classification since we wanted to apply an ‘Intent-to-Classify’ approach.

Step 2 was completely based on patient rated data and thus unaffected by therapist subjectivity. Data from patient self-rated measures was entered into the algorithm by the therapist (see Table 1 for details). The algorithm would then compare these data to cut-off values (see the appendix to Forsell et al. (2019b) for details) and for each patient give each variable a Green, Yellow (i.e. uncertain) or Red flag. Patients with only Green or only Red flags were classified as Green (given that Insomnia symptoms as measured by the ISI at week 3 was under 16) and Red respectively and did not go on to step 3. Patients with both Red and Green flags, or with only Yellow flags, where classified as Yellow and moved on to step 3.

In step 3, several other self-rated measures were included (see Table 1 for details). The cut-offs for measures that were already included in step 2 were changed in step 3 to be less restrictive and therefore give fewer yellow flags. Furthermore, a clinician-rated questionnaire was added were the clinician would rate the patient as Dark Red, Red, Yellow, Green or Dark Green on nine different domains, summarized briefly in Table 2. The rating was structured so that the clinician would read from lists with examples of things or situations that should lead to different ratings (see a full example of the domain Activity in the Appendix). Scores from these ratings were weighted differently depending on how central the domain was believed to be (see Table 2). The therapist scores were then combined with the automated flags from the patient ratings, and a final score was computed. If the score fell above a certain cut-off the patient would be classified as Green and vice versa for Red classification. There was however a Yellow area after step 3 as well. If the patient fell within this area, the clinician would decide on final classification together with an insomnia treatment expert (SJ and KB), which would then be labeled as Step 4.

**Table 2:** Clinician ratings for the RCT-Classification algorithm, with abbreviated examples.

Domain	Dark red	Dark green	Weight (+/−)
Activity in treatment (Activity)	Far behind schedule	Ahead of schedule	40
Contact in treatment (Contact)	Very little and/or mostly irrelevant contact	A lot of contact that is highly relevant	30
Sleep restriction and stimulus control (SRT)	Has not started SRT, started very incorrectly, is very negative	Has started SRT, with a high degree of understanding and initial success	60
Attitudes towards sleep and the CBT-i rationale (CBT-i)	Has a firm belief that contradicts the rationale	Has a view that is fully compatible with the rationale	30
Attitudes towards homework in general (Homework)	Very skeptical to homework, or misunderstands the homework	Very positive to homework and has done them correctly	30
Attitudes towards sleep medication (Sleep medication)^a^	Has a firm belief that taking sleep medication intermittently works well	Is very positive towards stabilizing, tapering and/or quitting sleep medication and this functions as a special motivator	20
Factors that may interfere with treatment adherence (Affected adherence)	Hindering factors, highly complicated life circumstances	No practical problems and very opportune life circumstances for going through the treatment at the moment	40
Factors that may interfere with sleep (Affected sleep)	Highly disturbed sleep from outside circumstances	Excellent sleep conditions	30
Patients own overtly expressed motivation (Motivation)	Clearly unmotivated	Clearly motivated	20

a In this domain, not taking sleep medication at all is given a Green rating.

### 3.3 Clinical outcome and prediction target

The primary outcome for the classifier in the RCT is the Balanced Accuracy of the predictions of treatment Failure, based on the Insomnia Severity Index (ISI). The ISI is a 7-item scale ranging from 0 to 28 points, that is reliable and sensitive to change (Bastien et al., 2001) and have established cut-offs for remission (<8 at post) and response (pre-post change >7) (C. M. Morin et al., 2011). We define Failure as being neither a responder nor a remitter (i.e. the patient did not improve substantially during treatment and is still a clinical case after treatment indicating that we may have been able to do more for the patient with more intensive care). Note: the use of the word Failure is not meant to indicate that the *patient* failed but instead that the treatment failed to help the patient.

#### 3.3.1 Classification accuracy measure

Balanced Accuracy is defined as BACC = (True Positives/All predicted Positives + True Negatives/All predicted Negatives)/2 (Brodersen et al., 2010). In this trial, BACC corresponds to (True Red/All Red + True Green/All Green)/2. It ranges from 0 to 1 where 0.50 is random and 1 is perfect. BACC is used because it a) compensates for uneven class distribution, b) is a single value, c) is expressible as a percentage, and c) is easy to understand. The terms Red is analogous with Positive, since we aim to identify failures. Thus, a True Positive classification is analogous with “True Red” (i.e. classified as Red and did indeed become a Failure). Ancillary accuracy measures such as sensitivity and specificity, calculated on a sample that has been reduced to adjust for uneven classes, which affect those measures, is presented in the Appendix.

### 3.4 Statistical analyses

#### 3.4.1 Accuracy of the RCT-classifier

The first aim was to establish the accuracy of the RCT-classifier used in the proof-of-concept study. To be useful, as a minimum, the classifier must be better than chance (i.e. have the lower bound of the 95 % confidence interval above 0.50). It is also important that all separate steps have a balanced accuracy that is better than chance, since if some steps do not, the contribution of such steps to the classifier as a whole could be cast in doubt. The results of the published RCT already suggest this classifier was good enough for clinical use but does not report a metric for the actual classification accuracy which is essential for future research and comparisons. Confusion matrix statistics was performed using the Caret package in R.

#### 3.4.2 Examination of the relative value of each of the predictors and the additive value of different logical sets of predictors

The second aim was to examine the relative value of each of the predictors for the classifier. To explore the content of the classification algorithm, we correlated Failure with the unadjusted values of all single predictors included in it, using the point biserial correlation. We also calculated cross-correlations between the predictors.

The third aim was to compare a larger number of prediction models by how much of the variance in outcome they can explain. We performed a series of logistic regressions including groups of predictors to examine their combined predictive power. Only predictors used by the RCT-classifier were included, in order to facilitate comparison between models. First, a model with all the patient ratings was be built, analogous with Step 2 in the RCT-classification algorithm (12 predictors). Secondly, a model with only the clinician ratings (9 predictors). Thirdly, a full model was built where all of the input predictors from the RCT-classifier were added to a single model (patient + clinician ratings = 21 predictors). Subsequently, a logistic regression was performed using only those predictors that had a significant correlation to the outcome Failure. This should make the model more parsimonious and require less data collection, making it a good comparator for the large model. Finally, the very simple model using only ISI measurements from screening until week three was added for comparison. This would be the simplest model to implement in another setting since weekly measurements of symptom levels are standard and would likely already be routinely collected.

The Veall and Zimmermann pseudo R^2^ for logistic regression will be used to compare models as it has been shown to be a close and robust estimation of the ordinary least squares r^2^ (Smith and McKenna, 2013; Walker and Smith, 2016), and is relatively stable across various base rates. The models were compared with each other using the Akaike Information Criterion (AIC) (Burnham and Anderson, 2004). AIC was compared using the formula Δ_*i*_ = AIC_*i*_ – AIC_min*,*_ where a Δ < 2 indicates strong support and Δ > 10 indicates essentially no support for model *i* compared to the best one of your models, and a Δ between 2 and 10 is acceptable.

#### 3.4.3 Missing data

Four (4) patients among those included in this study did not respond to the online post-treatment questionnaire. Three of these could be replaced with telephone-interview version of the ISI in accordance with (Hedman et al., 2013). Since only one participant out of 200 did not complete any post-treatment assessments (i.e. observed data is 99.5 %) no imputation or replacement of missing data was performed. Data for the predictor variables was mostly complete. Ten out of 21 predictors had any missing data. All but two of these were 98 % complete or more. The two variables with more missing was the MADRS-S mean variable, which was missing for 14 individuals (7 %) due to any missed weekly assessment and the knowledge-item which was missing for 15 individuals (7.5 %) for the same reason. None of the clinician rated predictor variables had any missing data. Since missing predictor values were very rare, they were handled with listwise deletion for the correlation and regression models.

## 4 Results

### 4.1 Aim 1: establishing the accuracy of the clinically useful RCT-classifier

Aim 1: The accuracy of the RCT-classifier algorithm in its entirety, as well as each classification step, are presented in Table 3. The final classification from the proof-of-concept study, which proved to be of clinical value, had a balanced accuracy of 67 % and its lower bound confidence interval was well above 50 % (chance). As can be seen in Table 3, most patients had to go to Step 3, which included therapist ratings, to be classified. The accuracies of Step 2 and 3 were significantly better than chance and thus likely both contributed to the classification as a whole, and no step was significantly better than any other step based on overlapping confidence intervals. However, the confidence interval for Step 1 is very wide and has its lower bound below 50 %. Only five patients were classified in this step, so even though four of them were classified correctly the very small sample makes the accuracy uncertain.

**Table 3:** Balanced accuracy of the classifier predicting Failure using the full sample (*n* = 199).

RCT-classifier	N=	True Red	False Red	True Green	False Green	Balanced Accuracy	95 % CI
Classified in Step1	5	3	1	1	0	0.75	0.37–1
Classified in Step2	25	10	7	8	0	0.77	0.60–0.93
Classified in Step3	165	16	12	101	36	0.60	0.53–0.68
Final Classification^a^	199	32	18	115	34	0.67	0.61–0.74

Notes: ISI=Insomnia Severity Index.

a Accuracy of RCT-classifier as used in Forsell et al., 2019b.

Several other indicators of accuracy are presented in Appendix A.

### 4.2 Aim 2: relative value of each of predictor and comparing predictive models

#### 4.2.1 Point-biserial correlations with failure

**Fig. 1:**
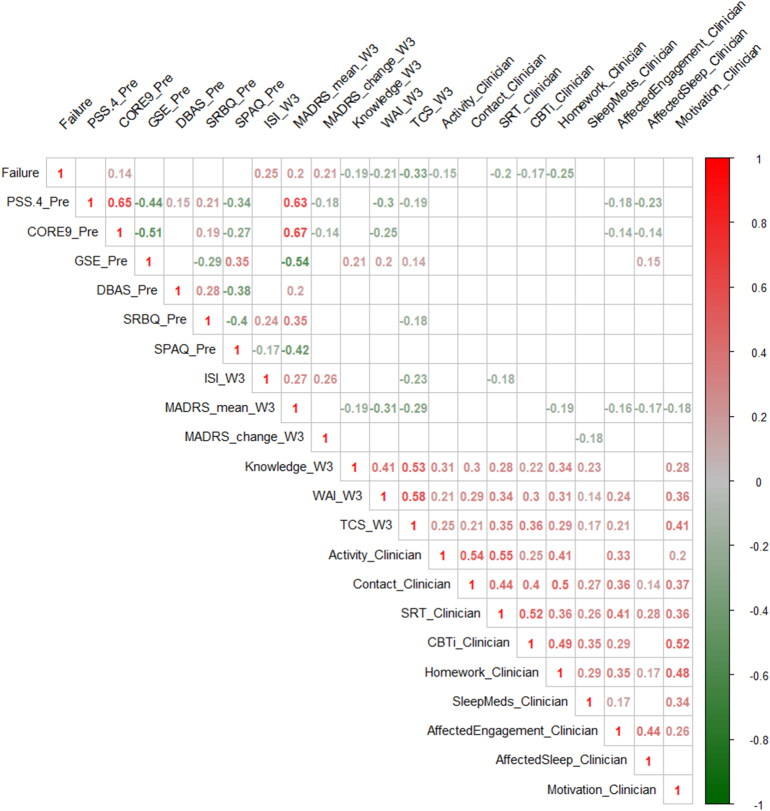
Correlations between predictors and Failure. *Notes: all coefficients displayed are significant at p* *<* *.05. Color saturation indicates strength of relationship (Red* *=* *Positive correlation with Failure, Green* *= Negative correlation with Failure).* *Pre* *= Data from start of treatment, W3* *=* *data from third week of treatment, CORE9* *= Clinical Outcomes in Routine Evaluation system-10 item scale with suicidality item removed, PSS.4* *=* *Perceived Stress Scale-4 item version, GSE* *=* *General Self Efficacy scale, DBAS* *= Dysfunctional Beliefs and Attitudes about Sleep, SRBQ* *=* *Sleep Related Behaviors Questionnaire, SPAQ* *= Sleep Problems Acceptance Questionnaire, MADRS* *=* *Montgomery-Åsberg Depression Rating Scale Self-report, WAI* *= Working Alliance Inventory, TCS* *= Credibility Expectancy Questionnaire, ISI* *= Insomnia Severity Index, Knowledge =* “To what extent have the things you've read about and done in treatment so far affected your knowledge about, and the way you think about, sleep and insomnia?”, *Clinician* *=* *Clinician rated data from third week of treatment, see*Table 2*.*

Fig. 1 shows the correlations between Failure and all predictors included in the RCT-classifier, as well as their cross correlations. From baseline, only CORE-9 correlated significantly with Failure, while all of the patient ratings from the treatment period (week 1–3) were significant, where the strongest correlation over all being treatment credibility (TCS). Out of the nine clinician ratings, activity in treatment (Activity), initiation and adherence of sleep restriction and stimulus control (SRT), understanding of and acceptance of the rationale (CBT—I), and attitudes towards homework (Homework) all correlated significantly.

#### 4.2.2 Aim 3: comparing different predictive models

Table 4 summarizes the results from the logistic regression models using the various predictors that were put into the classifier as well as a default model with only symptom ratings. The model using only variables with a significant correlation with Failure, had the lowest AIC (i.e. is AIC_min_ and Δ = 0). Compared to this, the full model will all data used in Forsell et al. (2019a) performs well. The model using all patient rating and the model using only symptom ratings perform acceptably, whereas the model using clinician ratings alone did substantially worse and had no support according to the AIC Δ. The full model had the highest *r*^2^, explaining 56 % of the variance in the odds of being a Failure. The model with all the patient ratings and the model with only significant predictors explained the same amount of variance (47 %), while the latter has a lower AIC. The clinician ratings only explained 16 % of the variance. However, the addition of these in the full model achieved 56 % variance explained as opposed to 47 % from just the patient ratings, suggesting some unique added information.

**Table 4:** Logistic regression using the data that was available to the RCT-classifier.

Model	Predictors	Predictors with sig. correlation with Failure (*p* < .05)	Significant predictors in model	AIC (Δ)	Veall Zimmerman *r*^2^
Patient ratings only	12	7	Stress⁎ Credibility⁎⁎ Depression change⁎	210.32 (2.5)	0.47
Clinician ratings only	9	4	Homework⁎⁎	253.71 (45.89)	0.16
Full (Patient+Clinician ratings) a	21	11	Stress⁎ Credibility⁎⁎⁎ Depression change⁎⁎ Homework⁎⁎ Sleep medication⁎	208.42 (0.6)	0.56
Only predictors with sig. Correlation with failure	11	11	ISI⁎ Credibility⁎⁎ Depression change⁎ Homework⁎	207.82 (AIC_min_)	0.47
Using only primary symptom measure (ISI from Screening through week 3)	5	2	NA	213.58 (5.8)	0.38

Notes: ISI = Insomnia Severity Index sum at week 3, Credibility = Treatment Credibility Scale (week 3), Homework = Clinician rating about patient attitudes to Homework in CBT (week 3), Stress = Perceived Stress Scale 4 item version (baseline), Depression change = Montgomery-Åsberg Depression Rating Scale-Self Report change from Pre to Week 3, Sleep medication = Clinician Rating about sleep medication use and willingness to taper or quit (week 3), AIC = Akaike information criterion (Δ < 2 means strong support, Δ = 2–10 means acceptable support and Δ > 10 means virtually no support).

a All input in RCT-classifier used in Forsell et al., 2019b.

⁎ *p* < .05.

⁎⁎ *p* < .01.

⁎⁎⁎ *p* < .001.

## 5 Discussion

In this study, we wanted to suggest an empirically supported minimal level of accuracy that a classifier predicting treatment failure for individual patients needs to reach in order to be potentially clinically useful, and to explore and compare prediction models of different complexity and implementation potential. We analyzed a classification algorithm previously used within a successful, i.e. clinically useful, Adaptive Treatment Strategy and found the Balanced Accuracy to be 67 % with a rather narrow confidence interval well above the level of chance.

This Balanced accuracy of 67 % could be used as a preliminary benchmark to decide if future classifiers of treatment outcome are good enough to be used early on in treatment to decide if the treatment should be adapted or not. However, some additional factors need to be considered. For example, the risks associated with adjusting, or not adjusting, treatment might differ between patient groups and types of treatments. However, it could be argued that it is reasonable to demand that the unadjusted treatment and the basic level of intensity or resources should be good enough to handle risks and the needs of the average patient. An indication of when a patient needs even more resources should still be helpful, and then the suggested benchmark is useful.

Another factor to consider in an Adaptive Treatment Strategy is if it is actually possible to adjust the treatment to avoid a predicted failure. The success of our case example was thus not only dependent on the ability to predict outcome, but also the fact that adjustment made a difference for patients risking failure. We studied internet-delivered treatment, where initial therapist contact was sparse but could easily be increased and standardized adjustments could be introduced quickly, as described in Forsell et al. (2019b). Other treatments or contexts might not allow this flexibility, or adjustments might not lead to better outcome, and then a good-enough predictive power is not necessarily enough for a successful application of an Adaptive Treatment Strategy.

A third factor that could play into deciding on the accuracy needed by a prediction is the costs associated with false positives and false negatives. False positives (i.e. identifying everyone as at-risk) would likely lead to better outcomes overall (more intensive treatment) but would be unachievable due to costs and limited resources. Conversely, the costs of false negatives could be that the patient will end up requiring more resources and longer treatment times overall. The algorithm evaluated here did not factor in how many patients we could “afford” to classify as Red nor how many misclassifications we could afford, but a more advanced algorithm surely could. This is an important area for future developments within adaptive treatment strategies.

With the above aspects in mind, the previous lack of gold standards for prediction accuracy in clinical decision-making within psychological treatments and psychiatry makes the results presented here an important step forward into a clinically validated and context appropriate benchmark for accuracy compared to older alternatives (Eisenberg and Hershey, 1983).

To further our understanding of the current classifier and explore alternatives, some in-depth analyses were performed. All steps in the classifier contributed above chance to the overall prediction, although the low statistical power for the patients classified in step 1 makes it impossible to draw firm conclusion. However, in the first step, four classifications out of five were correct, which suggests that it was possible to sort out “obvious cases” early on.

For the vast majority of patients, the classifier needed input from the therapist rather than using patient-rated data only. This consumes extra therapist time, which could ultimately limit scalability, and solutions using only patient-ratings are attractive.

Besides evaluating the input to the RCT-classifier, where predictors were selected, sometimes dichotomized, and combined, we also aimed to explore the predictive potential of specific predictors and of models combining different sets of predictors, by using regression analysis and comparing each model's level of explained variance, rather than the accuracies of actual classifiers.

For the 21 suggested predictors, we found that just over half of them had a significant correlation with treatment failure. When combined in a logistic regression, they (the “Full” model) explained the most variance (56 %) of all models. Only six predictors remained significant within the “Full” model, indicating that non-significant predictors still add to the model overall. However, this model does require a lot of data collection. It is also not parsimonious, could be prone to over-fitting, and is likely less robust than some of the simpler models since it contains less than five observed events (actual Failures) per predictor, a threshold which has been found to result in acceptable stability (Vittinghoff and McCulloch, 2006). On the other hand, the AIC for the “Full” model was relatively low. The model including only the eleven initially significant predictors (Significant only) achieved less explained variance (47 %), being equal to the model with all the patient ratings only. The “Significant only” model contained less noise as indicated by the lower AIC, but require four domain ratings from the clinician compared to none in the Patient-ratings-model. The model with only clinician ratings performed far worse, explaining only 16 % of outcome variance. However, the clinician ratings were intended to be complementary to the patient ratings, not a stand-alone model, and this is supported by the fact that the full model explained the most variance.

The regression model that used all patient ratings but without the dichotomizations used in the classification algorithm seems to perform better than the ISI-only model in terms of explained variance (47 vs 38 %), although the AIC values indicated that the models are very similar in terms of information loss. The overall impression is thus that a very basic model built on weekly self-rated symptoms, comparable to the one explored in (Forsell et al., 2019a), can perform fairly well, but additional self-ratings do increase predictive power.

To summarize the model comparisons, the full model with all ratings from patient and clinician explained the most variance. This indicates that clinician ratings did add unique information although they were rather weak as a stand-alone model. The probably most easily implemented model, using only weekly patient ratings of symptom severity, performed rather well and in line with similar models for other conditions (Forsell et al., 2019a) but still explained 10 % less than the other model that could be fully automated; the one where all patient-rated data was included. Adding more patient ratings and clinician ratings hence seems to provide additional predictive power compared to the very basic weekly measures model promoted by (Lambert, 2015), although this needs to be weighed against more time and effort spent by therapists and patients to provide predictive data.

From a clinical point of view, it is interesting to look further into the contribution by separate predictors. A general observation is that predictors measured early in treatment, rather than before treatment, are much more strongly associated with outcome. Insomnia severity at week three was naturally associated with Failure, and not surprisingly, levels of depression, stress and psychological distress in general were associated with worse outcomes. This fits with the assumption that general severity and complexity of patients is associated with lower success-rates, but none of these predictors was very strong, and other studies have found no clear connection between complexity and outcome of insomnia treatment (Morin et al., 2006).

The other significant factors we consider to be a combination of adherence and willingness, that could be conceptually summarized as treatment involvement; Activity, Treatment Credibility, Working Alliance, Homework (willingness and understanding that you will have to make actual changes), Sleep Restriction Therapy (doing as prescribed/willing), changes in Knowledge and understanding, willingness to taper Sleep medication (or not using it), and acceptance of the treatment rationale. This indicates that patients who, after several weeks of treatment, are actively skeptical to, refusing, or are simply not doing the treatment as prescribed, are less likely to benefit, much in line with a previous study on self-help treatment for Insomnia where treatment involvement with key treatment components related to better outcome (Kaldo et al., 2015b). The original randomized trial (Forsell et al., 2019b) indicates that these patients are far from lost causes but can achieve good outcomes if the therapists intensify their efforts and spend more time guiding the patient for the remainder of the treatment. It is important to note that despite the high face validity of these associations, establishing causal relationships between predictors and outcomes is beyond the scope of the current study. It is entirely possible that patients who display high treatment involvement are doing so because they are benefiting from treatment, as opposed to the other way around. The main aim of the current investigation was not to examine mechanisms of change, which would require different study designs.

Finally, we did not find correlations between measures of beliefs, acceptance and unhelpful behaviors related to sleep, but those were only measured at baseline and it is possible that changes in these factors would have been better predictors. It is also possible that the explanatory value of these predictors is overshadowed by insomnia symptoms during treatment.

### 5.1 Limitations

The foremost limitation in this study is that the sample size was chosen to detect clinically relevant group differences in an RCT, rather than for evaluating classification performance. However, the first aim – i.e. to create a benchmark for a good-enough predictive algorithm - was still met with a rather narrow confidence interval around the point estimate.

There is also a problem with the total number of the rarer of the two events Success or Failure (in this case number of Failures) in the sample, known in logistic regression as “the base rate”. A low base rate makes the logistic regressions less robust, and increases the need for replication in larger samples.

Another limitation is that we do not examine all available data, but only the data that was in fact used by the classification algorithm. The scope of the investigation was to examine the RCT-classifier and its parts, and it is reasonable to restrict the number of predictors to reduce instability, but it is possible that useful information was left out of the algorithm. Future studies should focus on investigating all available data and for example use machine-learning models that can handle such data (Boman et al., 2019).

Another limitation to the generalizability of our findings is that patients in the study are all self-referred, and then selected based on clinical presentation. Therefore, our conclusions need to be considered in the context of self-referred internet interventions within healthcare settings. This is the norm within the field of ICBT, but not necessarily within sleep research and other sleep related health care contexts in general.

### 5.2 Conclusions

We find that treatment failure in Internet-delivered Cognitive Behavior Therapy for Insomnia can be predicted with a balanced accuracy of 67 % four weeks into a nine-week long treatment using a relatively simple classification algorithm. This could serve as a preliminary, however much needed, minimum benchmark for when prediction accuracy could be clinically useful, since this classification algorithm was previously shown to be strong enough to fuel a successful Adaptive Treatment Strategy. Key predictive factors were symptoms of insomnia and depression and a range of factors related to treatment involvement, and future efforts in outcome prediction are encouraged to explore these types of factors further.

## Declaration of competing interest

The authors declare that they have no known competing financial interests or personal relationships that could have appeared to influence the work reported in this paper.

## Appendix A Supplementary data

Supplementary material Image 1

## Footnotes

1 Abbreviations: Akaike Information Criterion = AIC, Balanced Accuracy = BACC, Sleep Restriction Therapy = SRT, Insomnia Severity Index = ISI
