# Supplementary material for: Clinically sufficient classification accuracy and key predictors of treatment failure in a randomized controlled trial of Internet-delivered Cognitive Behavior Therapy for Insomnia
Source: Internet Interv. 2022 Jun 25;29:100554. doi: 10.1016/j.invent.2022.100554 (PMC9253627; doi:10.1016/j.invent.2022.100554)
Supplement: Supplementary file 1 — Supplementary material [file mmc1.docx]

**Appendix**

This appendix reports some complementary information and statistics as well as a brief overview of the treatment contents. In Table 1, a full example of the clinician rating for the domain Activity in treatment is presented. This can complement the abbreviated table in the main manuscript, which describes the same nine domains, but in a more compressed manner. Table 1 in this appendix is exactly as it was presented to the clinician when making the classification in the RCT (originally presented in Swedish).

**Table 1: Full example from Step 3 of the classification-algorithm: Clinician rating of the domain “Activity in treatment”**

| Color | Points | Criteria or examples |
| --- | --- | --- |
| Dark Red | -40 | Hasn’t started at all or just started with module 1 but there is a reasonable cause, which should no longer be an issue (temporary sickness, deadline at work) (if no reasonable cause exists, consider going back to step 1 and make immediate Red classification or in extreme cases exclude patient). Seems likely to be able to pick up speed and be more active.  OR  Only logged on a few times and maybe filled out some measures but nothing else  OR  Is only done with module 2-3^a^ and there’s no reasonable cause for the delay |
| Red | -20 | Finished module 2-3^a^ and with reasonable cause for delayed progress. Seems likely to be able to pick up pace and be more active OR  Finished module 4 or 5^a^ but has only done some of the work or has cut corners without reasonable cause |
| Yellow | 0 | Finished module 4^a^ diligently and with good reason for slow progress  OR  Finished module 5^a^ |
| Green | +20 | Finished module 5^a^ really meticulously, perhaps in spite of some resistance  OR  Halfway through module 6 and is working relatively well with content |
| Dark Green | +40 | Finished module 6 or more and working really well with content |

*^a^At the time of classification the patients are expected to be done with module 5, in which the patient should start and complete one week of sleep restriction therapy; the first three modules are very brief.*

**Ancillary accuracy measures for the classifier**

Table 2 presents some ancillary accuracy indicators from the RCT-classifier based on a reduced sample. Data from participants in the RCT who were classified as Red and then randomized to adapted treatment cannot be used to validate the classifier, as they have been manipulated between classification and outcome. To adjust for the imbalance in group size that was inevitably introduced by not using data from participants receiving adapted treatment in the RCT, in analyses where this imbalance would be a problem, half of the Green patients were also randomly removed (referred to as the adjusted sample). The primary outcome, Balanced Accuracy, is not meaningfully affected by this, but Sensitivity and Specificity for instance are.

**Table 2 Outcomes of classification based on reduced sample (n=125).**

|  | Adjusted sample  Final Classification |
| --- | --- |
| True Red | 32 |
| False Red | 18 |
| False Green | 19 |
| True Green | 56 |
| Sensitivity | .63 |
| Specificity | .76 |
| Positive Predictive Value | .64 |
| Negative Predictive Value | .75 |
| Precision | .64 |
| Recall | .63 |
| F1-score | .63 |
| Prevalence | .41 |
| Detection Prevalence | .40 |
| Balanced Accuracy (95% CI) | .69 (.61-.77) |

*Notes:* *Final classification = the actual outcome of the classification that was reached and used in the RCT by Forsell et al (2019a)*

Treatment description

The treatment is an Internet delivered Cognitive Behavior Treatment for insomnia (ICBT-i) based on a self-help book written by Dr. SJ (1) that has been adapted into an ICBT-format and previously tested in three randomized controlled trials (2-4). The basis of the treatment are the two primary components of CBT for insomnia: sleep restriction and stimulus control (5) which is the main focus of the entire treatment protocol used.

The treatment consisted of written material divided into modules (chapters) that were accompanied by worksheets and homework assignments as well as a sleep diary. The order of the modules was fixed in the standard treatment but could be altered for Red-Adapted patients once a new treatment plan was in place. Modules 1-3 were general introductory models and module 4 introduced the sleep restriction methodology. In module 4, patients should start sleep restriction. The patient was expected to finish modules 1-3 within a week and after that finish about one module per week. After module 5, patients are encouraged to engage with the remaining materials as a compliment to the sleep restriction and stimulus control during the remaining weeks.

The treatment was guided via written text-messages within the treatment platform by a licensed psychologist or supervised psychologist in training, who would review and give feedback on sleep diaries, homework assignments and symptom ratings as well as answer any questions the patient might have.

| Module | Title | Content |
| --- | --- | --- |
| 1 | Introduction | How the platform works, facts about sleep. What is sleep, what is it for? How much sleep do we need, and what consequences does sleep have? Is there such a thing as morning and evening people? What is insomnia? |
| 2 | The CBT-model | More about insomnia – how do we get insomnia and why does it not remit spontaneously? Introduction to cognitive behavioral therapy. Information about sleep medication and creating a cessation plan (optional) |
| 3 | Tired or sleepy-myths | Learning the difference between feeling tired and actually being sleepy/sleep deprived. Learning about the myths about sleep that may exacerbate insomnia. |
| 4 | Sleep rhythm | Introduction to sleep restriction and getting started |
| 5 | Bed-signals | Introduction to stimulus control and getting started |
| 6 | Daytime and bed-time | Routines for getting sunlight and exercise during the day and creating bed-time routines for consistency and winding down before bed |
| 7 | This is where you are | Acceptance, mindfulness, attitudes and expectations on sleep |
| 8 | Dealing with your thoughts | Cognitive reappraisal, focused on thoughts, rumination and worry about sleep |
| 9 | Sleep Hygiene- keep your sleep clean | Information and exercises about sleep hygiene such as avoiding caffeine later in the day, not drinking alcohol close to bedtime, keeping your bedroom cool and dark, and not having heavy meals at night |
| 10 | More thoughts, more acceptance | Some extra mindfulness materials and repetition of acceptance and cognitive reappraisal |
| 11 | So far and moving forwards | Summary and relapse prevention |

# References

1. Jernelöv S: Sov Gott – Råd och tekniker från KBT. Stockholm, Wahlström & Widstrand; 2008.

2. Kaldo V, Jernelöv S, Blom K, Ljótsson B, Brodin M, Jörgensen M, Kraepelien M, Rück C, Lindefors N. Guided internet cognitive behavioral therapy for insomnia compared to a control treatment – A randomized trial. Behaviour Research and Therapy. 2015;71:90-100.

3. Blom K, Jernelöv S, Kraepelien M, Bergdahl MO, Jungmarker K, Ankartjärn L, Lindefors N, Kaldo V. Internet Treatment Addressing either Insomnia or Depression, for Patients with both Diagnoses: A Randomized Trial. Sleep. 2015;38:267-277.

4. Blom K, Tillgren H, Wiklund T, Danlycke E, Forssén M, Söderström A, Johansson R, Hesser H, Jernelöv S, Lindefors N, Andersson G, Kaldo V. Internet-vs. group-delivered cognitive behavior therapy for insomnia: A randomized controlled non-inferiority trial. Behaviour Research and Therapy. 2015;70:47.

5. Harvey L, Inglis SJ, Espie CA. Insomniacs' reported use of CBT components and relationship to long-term clinical outcome. Behaviour Research and Therapy. 2002;40:75-83.
